# 6-Phosphofructo-2-kinase/fructose-2,6-bisphosphatase isoform 3 spatially mediates autophagy through the AMPK signaling pathway

**DOI:** 10.18632/oncotarget.20757

**Published:** 2017-09-08

**Authors:** Siyuan Yan, Xiaoli Wei, Shanshan Xu, Hui Sun, Weijun Wang, Ling Liu, Xuejun Jiang, Yongxiang Zhang, Yongsheng Che

**Affiliations:** ^1^ State Key Laboratory of Mycology, Institute of Microbiology, Chinese Academy of Sciences, Beijing 100101, China; ^2^ State Key Laboratory of Toxicology & Medical Countermeasures, Beijing Institute of Pharmacology & Toxicology, Beijing 100850, China; ^3^ University of Chinese Academy of Sciences, Beijing 100039, China

**Keywords:** PFKFB3, glycolysis, nuclear localization, autophagy, AMPK

## Abstract

6-Phosphofructo-2-kinase/fructose-2,6-bisphosphatase isoform 3 (PFKFB3), is a critical enzyme for glycolysis and highly expressed in cancer cells. It plays an essential role in regulating metabolism, angiogenesis, and inflammation. Although PFKFB3 is involved in modulating autophagy, its regulatory role appears to be either positive or negative, which remains to be clarified. Unlike other PFK-2/FBPase isoforms, PFKFB3 can localize in both nucleus and cytoplasm, leading to the speculation that subcellular localization of PFKFB3 may play a regulatory role in autophagy. Here, we found that either a PFKFB3 inhibitor or PFKFB3 silencing by siRNA, suppressed the basal and the H_2_O_2_-induced autophagy concomitantly with the inhibition of AMPK activity. While overexpression of the wild type PFKFB3 promoted the H_2_O_2_-induced autophagy, the K472/473A mutated PFKFB3, which lost nuclear localizing property, inhibited the autophagic process. Although the K472/473A mutated PFKFB3 stimulated more lactate production, it decreased the activity of AMPK compared to the wild type PFKFB3. Moreover, PFKFB3 similarly regulates the autophagy induced by rasfonin, a fungal secondary metabolite that downregulates the activity of AMPK. Compound C, a widely used AMPK inhibitor, induced the autophagic process but reduced the H_2_O_2_-dependent autophagy. Collectively, the data demonstrated that PFKFB3 localizing in nucleus is essential for its regulatory role in autophagy, and PFKFB3 at least positively regulated the H_2_O_2_-induced autophagy through the AMPK signaling pathway, which likely played dual roles in the process.

## INTRODUCTION

Metabolism of cancer cells supports continuous cell growth and proliferation in tumor microenvironment [1]. Cancer cells are prone to high rate of aerobic glycolysis instead of oxidative phosphorylation even in the presence of oxygen, a phenomenon known as the Warburg effect [2], which is also typical for many nontransformed cells during rapid proliferation. The main function of glycolysis is to provide anabolic substrates, not the ATPs, in the rapidly proliferating cells [3, 4]. Although energy production in glycolysis is less efficient than oxidative phosphorylation, the enhanced glycolysis provides essential metabolic intermediates important for cell growth and survival.

In the three rate-limiting enzymes (hexokinase, PFK-1, and pyruvate kinase) involved in glycolysis [5], PFK-1 catalyzes the conversion of fructose 6-phosphate to fructose-1,6-bisphosphate (F1,6BP) and serves as a gatekeeper in metabolic degradation of glucose. PFK-1 is controlled by the downstream metabolites, mainly by its allosteric activator, fructose-2,6-bisphosphate (F2,6BP), which is synthesized and degraded under the regulation of a bifunctional enzyme, 6-phosphofructo-2-kinase/fructose-2,6-bisphosphatase (PFK-2/FBPase) [6]. Several PFK-2/FBPase-2 isoenzymes encoded by four different genes, *PFKFB1* to *PFKFB4*, have been characterized in mammals [7], and showed distinct regulatory and kinetic properties (kinase and phosphatase activities). PFKFB3 is known to have the highest ratio (>700) of kinase/phosphatase activity to maintain the elevated level of F2,6BP, thereby sustaining a high rate of glycolysis [1, 8]. It is highly expressed in tumor, endothelial, and the activated immune cells, and plays an essential role in regulating metabolism, angiogenesis, and inflammation [1, 9]. Meanwhile, it can be induced by progesterone, inflammatory stimuli, and hypoxia. Unlike other PFK-2/FBPase isoforms, PFKFB3 can localize in both cytoplasm (the site of glycolysis) and nucleus, and the nuclear PFKFB3 increases cell proliferation and the expression of several key cell cycle proteins, but decreases the expression of cell cycle inhibitors [10]. It has been reported that the inhibition of PFKFB3 promoted autophagy in HCT116 cells [11], while knockdown of PFKFB3 restrained the activation of autophagy in rheumatoid arthritis T cells [12]. Thus, the role of PFKFB3 in regulating autophagy remains unclear and controversial.

Macroautophagy (hereafter called autophagy) is an evolutionarily conserved catabolic, intracellular membrane trafficking process involved in the delivery of cytoplasmic contents and organelles to lysosomes for degradation [13]. It is induced under various conditions, such as amino acids starvation and energy stress [14]. In eukaryotic cells, the mammalian target of rapamycin (mTOR) and the AMP-activated protein kinase (AMPK) signaling pathways are essential regulators of autophagy [15, 16]. In addition, mTOR complex-1 (mTORC1), activated by amino acids and blocked by rapamycin (Rapa) [17], is one of the key sensors for amino acids in mammal cells, whereas AMPK, a member of the serine/threonine protein kinases, is a highly conserved sensor for the intracellular adenosine nucleotides and plays a key role in regulating energy homeostasis [18]. The phosphorylation of a conserved threonine residue (Thr172) in the catalytic α-subunit is thought to be prerequisite for the activity of AMPK [19]. Several AMPK upstream activating kinases have been identified, such as LKB1 (liver kinase B1), CaMKK2 (Calcium/Calmodulin-dependent Protein Kinase Kinase 2), and TAK1 (transforming growth factor *β*-activated kinase-1). It is well known that various cellular stresses can activate AMPK. Adenosine monophosphate (AMP) and diphosphate (ADP) inhibit Thr172 dephosphorylation and promote the activity of AMPK, whereas adenosine triphosphate (ATP) shows the opposite effect [20]. In addition to physiological AMP/ADP elevation resulted from the stresses, such as low nutrients or prolonged exercise, pharmacological agents including 5-aminoimidazole-4-carboxamide1-*β*-d-ribofuranoside (AICAR) and metformin, can also activate AMPK [21]. And compound C (CC) is a widely used ATP-competitive inhibitor of AMPK [21]. Regarding autophagic regulation, AMPK can activate autophagy by deactivating its negative regulator mTORC1 [22]. Recently, AMPK was found to mediate autophagy by direct phosphorylation of its initiator Ulk1, a serine/threonine kinase and the mammalian functional homolog of the yeast ATG1 [15, 23]. Thus, AMPK could activate autophagy via a “double insurance” mechanism. However, AMPK was also reported to inhibit autophagy in isolated rat hepatocytes [24]. Therefore, AMPK plays bidirectional roles in autophagy regulation.

In the current work, we have proved that inhibition of PFKFB3 not only inhibited the basal autophagy, but also blunted the rasfonin- or H_2_O_2_–upregulated autophagic flux. Furthermore, PFKFB3 showed nuclear localizing property, and its regulatory roles in autophagy were subcellular localization-dependent and associated with the AMPK signaling pathway.

## RESULTS

### PFK-15 inhibits the autophagic processes

**Figure 1:**
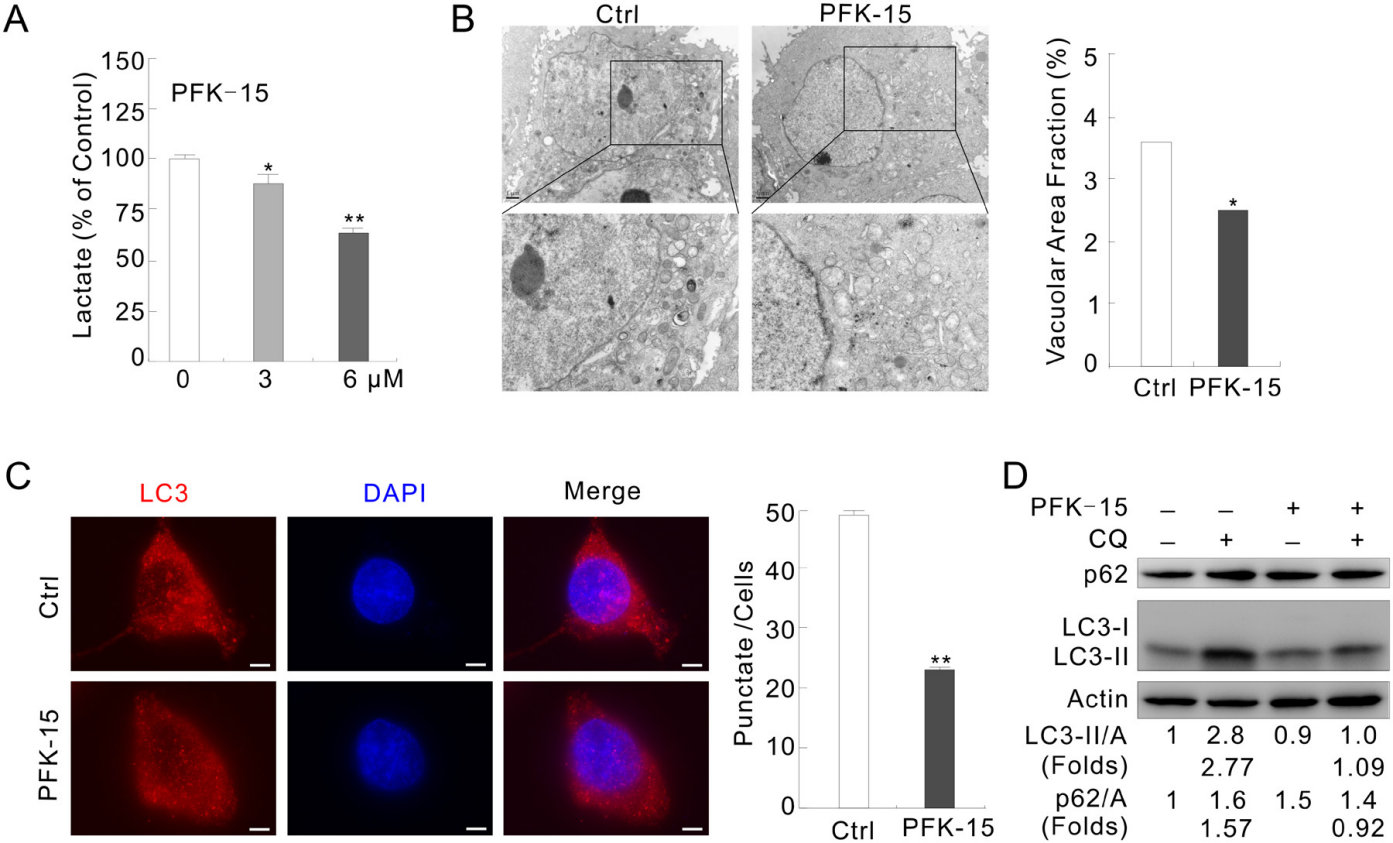
PFKFB3 inhibitor PFK-15 reduces autophagy in ACHN cells **(A)** ACHN cells were treated with PFK-15 (0–6 μM) for 12 h, and the secreted lactate was analyzed. **(B)** Electron microscopy was performed following treatment with PFK-15 (6 μM unless otherwise indicated) for 2 h. Morphometric analysis of the area fractions between autophagosomes and cytoplasm were calculated using ImageJ software. The data of area ratios were non-normally distributed and presented as the mean of at least 20 cells counted for each group. **(C)** Immunofluorescence using the LC3 antibody was performed following treatment with PFK-15 for 2 h. Numbers of the punctate LC3 in each cell were counted, and at least 30 cells were included for each group. Bar = 10 μm. **(D)** Following treatment with PFK-15 in the presence or absence of 10 μM CQ for 2 h, cell lysates were prepared and analyzed by immunoblotting using the indicated antibodies. For histogram results, the data were presented as mean ± S.D. and analyzed by T-test. *P < 0.05 vs control; **P < 0.01 vs control.

To clarify the function of PFKFB3 in autophagy regulation, PFK-15, a small molecule antagonist of PFKFB3 [25], was used and the aroused autophagy was monitored. Since renal cell carcinoma (RCC) is a model to study the role of an oncologic-metabolic shift (commonly referred as the “Warburg effect”) leading to malignancy [26], renal cancer cell line ACHN was selected for this study. Considering the fact that treatment of ACHN cells with 6 μM PFK-15 significantly decreased the level of secreted lactate (final product of glycolysis) at the time of detection (Figure 1A), a concentration of 6 μM was also used for PFK-15 in the following experiments.

Under transmission electron microscopy (TEM) [27], formation of autophagosomes was reduced and the area ratios of autophagosomes to whole cells were significantly decreased in the PFK-15-treated cells (Figure 1B). In immunofluorescence assay, we found that PFK-15 treatment decreased the punctate staining of LC3, the mammalian homolog of yeast Atg8 (Figure 1C). While immunoblotting results revealed that PFK-15 decreased the level of LC3-II but increased the amount of autophagy substrate p62 (Figure 1D). Chloroquine (CQ), a known inhibitor of autolysosome degradation widely used in monitoring autophagic flux [28], failed to accumulate LC3-II and block p62 degradation in the PFK-15-treated cells (Figure 1D). In HeLa cells, PFK-15 also completely inhibited autophagy as evidenced by LC3-II accumulation and p62 degradation in the presence of CQ (Supplementary Figure 1A). Furthermore, bafilomycin A1 (Baf-A1), which blocks the fusion of autophagosomes and lysosome [28], showed similar effect to CQ on the PFK-15-regulated autophagy in both HeLa and ACHN cells (Supplementary Figure 1B and 1C). These results indicated that PFK-15 inhibits the basal autophagy.

### PFK-15 attenuates autophagy upon stimulation with either rasfonin or H_2_O_2_

**Figure 2:**
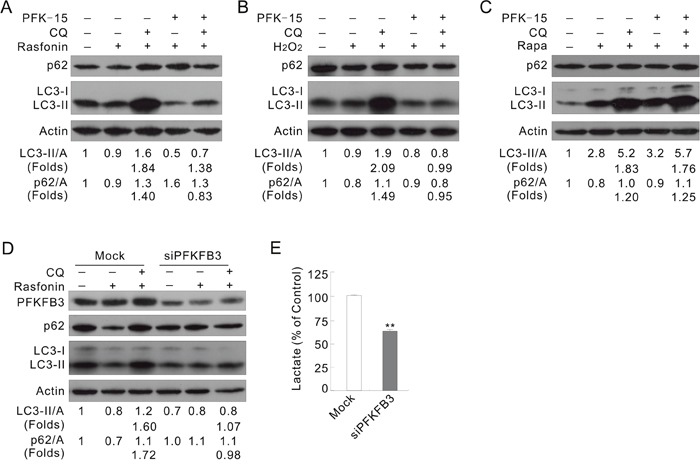
Inhibition of PFKFB3 attenuates the rasfonin-induced autophagy **(A)** ACHN cells were treated with 6 μM rasfonin or a combination with PFK-15 in the presence or absence of 10 μM CQ for 2 h. Cell lysates were prepared and analyzed by immunoblotting. **(B and C)** ACHN cells were treated with H_2_O_2_, or 0.1 μM Rapa or a combination with PFK-15 in the presence or absence of CQ for 2 h. **(D and E)** ACHN cells were transfected with PFKFB3 or the Mock control siRNAs for 48 h. The lysates were analyzed by immunoblotting following treatment with 6 μM rasfonin for 2 h in the presence or absence of 10 μM CQ (D). Suspension was collected before drug treatment and lactate assay was performed (E), and double asterisk means p < 0.01.

Rasfonin, a natural product originally isolated from the fungus *Talaromyces* sp. 3656-A1 and named for its activity against the small G-protein Ras, has been demonstrated to promote the autophagic process in our previous study [29]. Here it was found to cause obvious accumulation of membrane vacuoles in a time-dependent manner (Supplementary Figure 2A) and to affect the ratios of LC3-II/actin in a duration-dependent manner, but to promote p62 degradation at all time points by immunoblotting (Supplementary Figure 2B). While rasfonin increased the ratios of LC3-II/actin at both the 1 and 12 h time points, it decreased the level of LC3-II at either 2 or 4 h treatment (Supplementary Figure 2C and 2D). However, CQ blocked the rasfonin-induced downregulation of LC3-II or p62 at all the time points tested (Supplementary Figure 2C and 2D), suggesting an enhanced autophagic flux. The aforementioned results showed that rasfonin promoted lysosomal turnover of endogenous LC3, an autophagy marker [30], and could be used as an autophagy inducer. Treatment of ACHN cells with a combination of rasfonin and PFK-15 accumulated less LC3-II in the presence of CQ (Figure 2A; folds: lane 5 vs 3), and failed to degrade p62 compared to that with rasfonin alone (Figure 2A; lane 4 vs 2). It is generally accepted that autophagy regulation is not simply in a “either promotion or inhibition” pattern, inhibition to certain extent is usually observed [31]. In the PFK-15/rasfonin treated cells, CQ still accumulated LC3-II, suggesting occurrence of an incompletely blocked autophagic process. In fact, CQ failed to increase the level of p62 in the PFK-15/rasfonin treated cells (Figure 2A). Here, “changed folds” was used to indicate the magnitude in changes for the levels of LC3-II/Actin and p62/Actin compared to that without CQ, which represent the intensity of autophagic flux. Treatment of HeLa cells with PFK-15 completely blocked the rasfonin-induced autophagy, as evidenced by accumulation of LC3-II and degradation of p62 in the presence of CQ (Supplementary Figure 3A; folds: lane 5 vs 3).

Reactive oxygen species (ROS), highly reactive oxygen free radical or non-radical molecules produced by multiple mechanisms [32], has been demonstrated to promote the starvation-induced autophagy, antibacterial autophagy, and autophagic cell death [33]. As a donor of ROS, H_2_O_2_ was widely used as an autophagy inducer [34]. Here, we observed that H_2_O_2_ alone induced autophagic flux, but PFK-15 completely inhibited this process (Figure 2B; folds: lane 5 vs 3). However, PFK-15 failed to attenuate the Rapa-induced autophagy as CQ normally blocked the autophagic flux in the PFK-15/Rapa-treated cells (Figure 2C; folds: lane 5 vs 3). These results indicated that that PFK-15 regulates autophagy in a stimulus type-dependent manner.

### Deprivation of PFKFB3 attenuates the rasfonin- and H_2_O_2_-induced autophagy

To verify the results obtained from PFK-15 treatments, PFKFB3 in ACHN cells was genetically deprived using target siRNA. Deprivation of PFKFB3 decreased the basal level of LC3-II, and treatment with rasfonin accumulated less LC3-II in the presence of CQ (folds: lane 6 vs 3) and failed to degrade p62 (lane 5 vs 2) in the PFKFB3-depleted cells (Figure 2D). Similar results were also obtained in HeLa cells (Supplementary Figure 3B). In the H_2_O_2_-treated cells, loss of PFKFB3 decreased the autophagic flux (Supplementary Figure 3C; folds: lane 3 vs 6), while silencing of PFKFB3 significantly decreased secreted lactate in ACHN and HeLa cells (Figure 2E and Supplementary Figure 3D). However, deprivation of PFKFB3 in ACHN cells failed to attenuate the Rapa-induced autophagy (Supplementary Figure 3E).

### The K472/473A mutant fails to localize in nucleus

**Figure 3:**
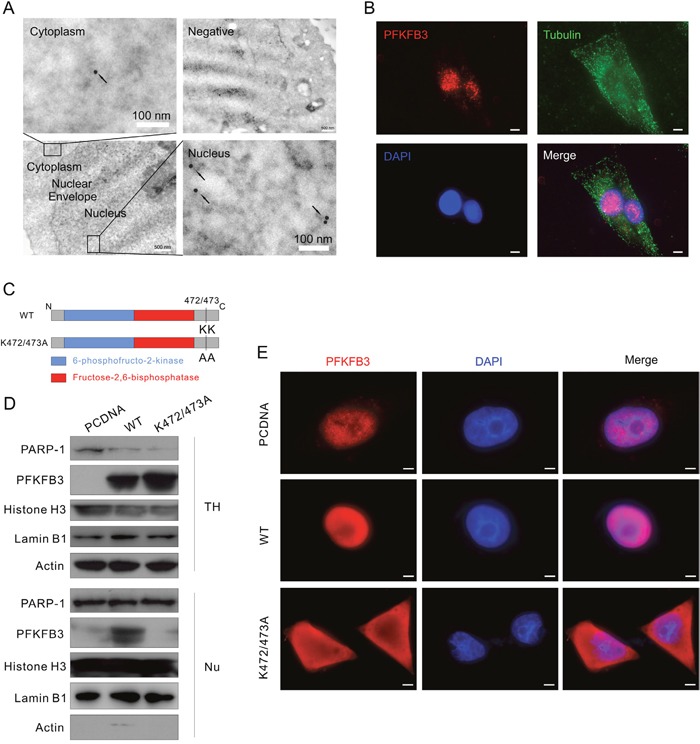
K472/473A mutation changes the nuclear localization of PFKFB3 **(A)** HeLa cells were analyzed by immunoelectron microscopy using anti-PFKFB3 antibody. Images are representative of at least 20 cells. Arrowheads, PFKFB3 signals (gold particles). The negative control was prepared without addition of specific antibody. **(B)** Immunofluorescence was performed for ACHN cells using the antibodies of PFKFB3 and Tubulin, and the nuclei were stained by DAPI. Bar = 20 μm. **(C)** Schematic diagram of WT PFKFB3 (top) and K472/473A mutated PFKFB3 (bottom). **(D and E)** After transfection with the PCDNA, WT or K472/473A plasmids, TH and Nu subcellular fractions were extracted from ACHN cells and analyzed by immunoblotting with the antibodies indicated (D); cells were stained using the PFKFB3 antibodies and the nuclei were stained by DAPI (E). Bar = 10 μm. Images were representative of at least 20 cells.

Since PFKFB3 has been reported to localize in the nuclei of HeLa cells [10], subcellular localization of endogenous PFKFB3 was examined in HeLa and ACHN cells. Distribution of PFKFB3 in HeLa cells was investigated by immunoelectron microscopy, and dots of immunogold were observed in both nuclei and cytoplasm (Figure 3A). In immunofluorescence assay, PFKFB3 mainly localized in the nuclei of ACHN and HeLa cells as observed by either fluorescence microscopy or confocal microscopy (Figure 3B and Supplementary Figure 4A). To further determine its localization, the protein expression in total homogenate (TH) and the nuclear fractions (Nu) extracted from ACHN cells were examined. As expected, the expression of mainly PFKFB3 was detected in the nuclear fractions (Supplementary Figure 4B), using actin and PARP-1 (poly ADP-ribose polymerase; an enzyme involved in DNA repair) as the markers for TH and Nu [35], respectively. Meanwhile, either Lamin B1 or histone H3, two often used nuclear markers [36, 37], was also detected (Supplementary Figure 4B).

The structural organization of PFKFB3 is similar to other isoenzymes, with the kinase and bisphosphatase domains located in the center flanked by regulatory regions (Figure 3C). Since the K472 and K473 lysine residues at the carboxyl terminal are essential for nuclear targeting [10], they were replaced by alanine residues using site-directed mutagenesis (Figure 3C). The expression and distribution patterns of the K472/473A mutated PFKFB3 were confirmed by immunoblotting after subcellular fractionation (Figure 3D). Except for the difference in fluorescence intensity, distribution of PFKFB3 remained nearly unchanged, localizing in the nuclei of either the PCDNA- or WT-transfected cells, whereas the K472/473A mutated PFKFB3 mainly localized in cytoplasm (Figure 3E; exposure time: 800 ms for PCDNA, 100 ms for WT and K472/473A). Similar results were also obtained in HeLa cells (Supplementary Figure 4C). Taken together, the K472/473A mutation of PFKFB3 altered its nuclear localization.

### Overexpression of the K472/473A mutated PFKFB3 partially inhibits autophagy

**Figure 4:**
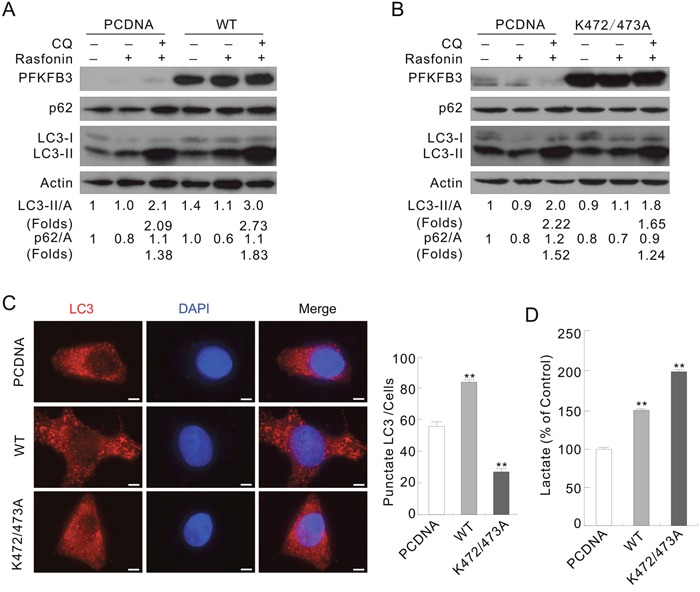
The WT and K472/473A mutated PFKFB3s play different roles in regulating autophagy ACHN Cells were transfected with the indicated plasmids for 36 h. **(A and B)** Following treatment with 6 μM rasfonin in the presence or absence of 10 μM CQ for 2 h, cell lysates were analyzed by immunoblotting with the antibodies indicated. **(C)** LC3 antibody was used to label the endogenous LC3, and DAPI was used to stain the nuclei. Pictures were taken by fluorescence microscopy. Numbers of the punctate LC3 in each cell were counted, and at least 30 cells were included for each group. Bar = 10 μm. **(D)** Suspension was collected before the treatment and lactate assay was performed. Double asterisk means p < 0.01.

To investigate whether PFKFB3 spatially regulate autophagy, ACHN or HeLa cells were transfected with the plasmids carrying either the WT or K472/473A mutated PFKFB3. Overexpression of the WT, not the K472/473A mutated PFKFB3, enhanced the autophagy upon rasfonin stimulation in ACHN cells by evaluating LC3-II accumulation and p62 degradation in the absence or presence of CQ compared to the PCDNA-control cells (Figure 4A and 4B; folds: lane 6 vs 3). The basal level of LC3-II was increased in WT PFKFB3-, but not K472/473A mutated PFKFB3-transfected cells. Similarly, WT PFKFB3 promoted the rasfonin-induced autophagic flux, and K472/473A mutated one partially attenuated this process in HeLa cells (Supplementary Figure 5A; lane 3 vs 6 vs 9). In addition, overexpression of the WT PFKFB3 promoted the H_2_O_2_-dependent autophagy (Supplementary Figure 5B; lane 6 vs 3), whereas the K472/473A mutant partially inhibited the induced autophagy (Supplementary Figure 5B; lane 9 vs 3). Here, the induced rather than the basal autophagy was the focus of our study, and inconsistency between the LC3-II levels of the Mock- and PCDNA-control cells was indeed noticed upon individual treatment with rasfonin or H_2_O_2_, which could be resulted from the alteration in cell condition after transfection [28, 30]. Therefore, the autophagic flux instead of the level of LC3-II was analyzed in all the experiments. The punctate staining of LC3 was obviously increased in the WT PFKFB3, but not the K472/473A-transfected cells without any treatment in immunofluorescence assay (Figure 4C). Interestingly, both the WT and K472/473A mutated PFKFB3 promoted lactate secretion, and overexpression of the latter generated more lactate (Figure 4D), which is in agreement with the report that *N*-methyl-d-aspartate subtype of glutamate receptors (NMDAR)-induced cytosol localized PFKFB3 increased the rate of glycolysis [38]. These results indicated that nuclear localization of PFKFB3 plays a critical role in regulation of autophagy, but whether lactate is involved in the process remained uncovered.

### AMPK is involved in the PFKFB3-regulated autophagy

**Figure 5:**
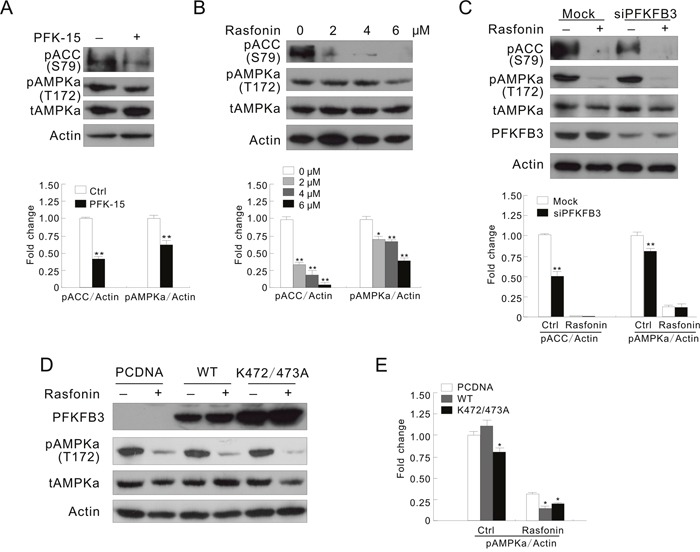
AMPK is involved in the PFKFB3-regulated autophagy process **(A)** ACHN cells were treated with PFK-15 for 2 h. **(B)** ACHN cells were treated with 0-6 μM rasfonin for 2 h. **(C)** After transfection with PFKFB3 or the Mock control siRNAs for 48 h, ACHN cells were treated with 6 μM rasfonin for 2 h. **(D and E)** Following transfection with the indicated plasmids for 36 h, ACHN cells were treated with 6 μM rasfonin for 2 h. Cell lysates were prepared and analyzed by immunoblotting using the indicated antibodies. Quantification of the signals were shown in the bar graphs (n = 3). *P < 0.05 vs control, **P < 0.01 vs. control.

AMPK, a sensor for intracellular adenosine nucleotides, responds to the changes in energy status and coordinates cell growth, autophagy, and metabolism [18]. Since it generally phosphorylates PFKFB3 at Ser461, its role in the PFKFB3-mediated autophagy was explored. Treatment with PFK-15 alone decreased the phosphorylation of AMPKα and ACC (acetyl-CoA carboxylase) at Ser79, a known substrate of AMPK [39], suggesting that PFK-15 inhibited the activity of AMPK in both ACHN and HeLa cells (Figure 5A and Supplementary Figure 6A). Moreover, PFK-15 reduced the phosphorylation of both AMPKα and ACC upregulated by H_2_O_2_ stimulation (Supplementary Figure 6B). However, different from treatment with H_2_O_2_, rasfonin treatment induced autophagy concurring with inhibition of both the phosphorylation and activity of AMPKα (Figure 5B). In addition, deprivation of PFKFB3 reduced the basal and the induced phosphorylation of ACC, suggesting that loss of PFKFB3 attenuated the activity of AMPK (Figure 5C and Supplementary Figure 6C). Although rasfonin and H_2_O_2_ have been demonstrated to oppositely regulate the activity of AMPK, deprivation of PFKFB3 decreased the phosphorylation of AMPKα in response to stimulation with either rasfonin or H_2_O_2_ (Figure 5C and Supplementary Figure 6C), and loss of PFKFB3 obviously suppressed the H_2_O_2_-induced phosphorylation of ACC (Supplementary Figure 6C). Therefore, we speculated that PFKFB3 might function upstream of AMPK. In contrast to the K472/473A-transfected cells, overexpression of the WT PFKFB3 increased the H_2_O_2_-induced phosphorylation of ACC and AMPKα, suggested the enhanced AMPK activity (Supplementary Figure 6D and 6E). However, the K472/473A mutated PFKFB3 decreased the phosphorylation of AMPKα in untreated, and rasfonin- and H_2_O_2_-treated ACHN cells (Figure 5D and 5E; Supplementary Figure 6D and 6E). Considering that WT PFKFB3 differently regulated the phosphorylation of AMPKα in the rasfonin- and H_2_O_2_-treated cells, but promoted the induced autophagy, PFKFB3 regulates autophagy through multiple pathways including AMPK, and AMPK may play a dual role in regulation of autophagy in the same cells.

### Bidirectional roles of AMPK in regulating autophagy

**Figure 6:**
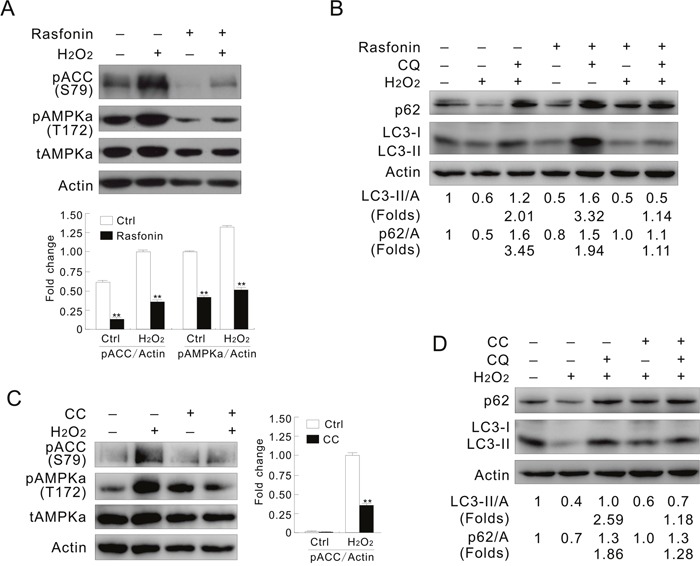
Inhibition of AMPK mimics the effect of inhibition of PFKFB3 **(A and B)** ACHN cells were treated with 0.1 mM H_2_O_2_ or a combination with 6 μM rasfonin in the presence or absence of 10 μM CQ for 2 h. **(C and D)** ACHN cells were treated with 0.1 mM H_2_O_2_ or a combination with 20 μM CC in the presence or absence of 10 μM CQ for 2 h. Cell lysates were prepared and analyzed by immunoblotting using the indicated antibodies. Quantification of the signals were shown in the bar graphs (n = 3). **P < 0.01 vs. control.

Despite the fact that rasfonin decreased the activity of AMPK, it also blunted the H_2_O_2_-promoted phosphorylation of AMPKα/ACC levels, and inhibited the H_2_O_2_-dependent autophagic flux (Figure 6A and 6B), suggesting involvement of AMPK in the process. To prove the hypothesis, a widely used inhibitor of AMPK, CC was used in the experiments [21]. Similar to previous study, in which CC inhibited the activity of AMPK independent of phosphorylation, but decreased the activity of its downstream molecule [40], CC was found to increase the basal phosphorylation of AMPKα (Figure 6C). Nevertheless, decrease in both the basal and the H_2_O_2_-induced phosphorylation of ACC suggested that CC definitely inhibited the activity of AMPK (Figure 6C). Consistent with that reported previously [41], treatment with CC alone increased the autophagic flux (Supplementary Figure 7A). However, the H_2_O_2_-induced autophagic flux was reduced by CC since the fold changes were remarkably decreased in the H_2_O_2_/CC-treated ACHN cells (Figure 6D). On the contrary, CC further enhanced the autophagic flux induced by rasfonin (Supplementary Figure 7B; folds: lane 5 vs 3). Different from CC treatment, the AMPK agonist AICAR further increased the H_2_O_2_-induced phosphorylation of AMPKα and ACC (Supplementary Figure 7C), and promoted the basal and H_2_O_2_-dependent autophagy (Supplementary Figure 7D; folds: lane 7 vs 3). These results indicated that AMPK is indeed required to activate the autophagic process in response to H_2_O_2_ challenge, but negatively regulates the rasfonin-induced autophagy. Thus, it played a dual role in regulating autophagy depending on the type of stimulus.

### Deprivation of AMPKα differently regulates the H_2_O_2_– and rasfonin-induced autophagy

**Figure 7:**
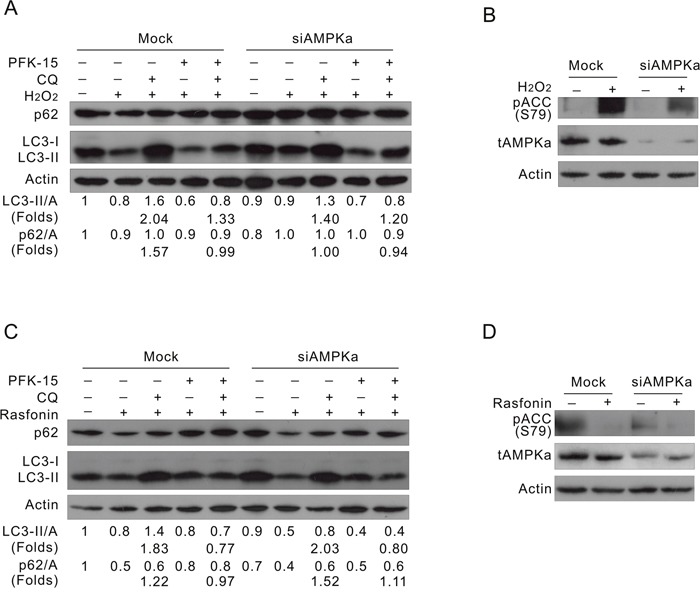
Deprivation of AMPKα suppresses the H_2_O_2_-, but not rasfonin-induced autophagy ACHN cells were transfected with the AMPKα1/2 siRNA for 48 h. **(A and B)** Cells were treated with 0.1 mM H_2_O_2_, with or without PFK-15 in the presence or absence of 10 μM CQ for 2 h. **(C and D)** Cells were treated with 6 μM rasfonin, with or without PFK-15 in the presence or absence of 10 μM CQ for 2 h. Cell lysates were prepared and analyzed by immunoblotting using the indicated antibodies.

To further investigate the role of AMPK in the PFKFB3-mediated autophagy, AMPKα1/2 was knocked down in ACHN cells by target siRNA. Interestingly, deprivation of AMPKα1/2 inhibited the induced autophagy (Figure 7A; lane 8 vs 3) concurring with the reduced phosphorylation of ACC in response to H_2_O_2_ challenge (Figure 7B). The basal level of LC3-II was also slightly decreased in the AMPKα1/2-depleted cells (Figure 7A; lane 6 vs 1). Notably, PFK-15 suppressed the H_2_O_2_-induced autophagy in the Mock-control cells (Figure 7A; lane 5 vs 3), and displayed less inhibitory effect on the H_2_O_2_-induced autophagy in the AMPKα1/2-depleted cells (Figure 7A; lane 10 vs 8). Since PFK-15 still showed inhibitory effect on the H_2_O_2_-induced autophagy in the AMPKα1/2-depleted cells, we speculated that an effector other than AMPKα might function downstream of PFKFB3 to regulate the induced autophagic process. While PFK-15 inhibited the induced autophagy in the Mock-control or AMPKα1/2-depleted cells (Figure 7C; lane 5 vs 3 and lane 10 vs 8), deprivation of AMPKα1/2 failed to attenuate the rasfonin-induced autophagic flux (Figure 7C and 7D; lane 8 vs 3), which is consistent with the fact that rasfonin induced autophagy accompanied by decreased phosphorylation of AMPKα. Thus, we assumed that PFKFB3 and AMPK might function in the same regulating axis to mediate autophagy, and PFKFB3 exerts its regulatory effect at least partially through AMPK under certain circumstances. Taken together, PFKFB3 regulated autophagy depending on its nuclear localization and relating to the AMPK signaling.

## DISCUSSION

A new finding of the present study is that the nuclear localization of PFKFB3 plays an essential role in regulating autophagy. Apparently, PFKFB3 regulates autophagy in a stimulus-dependent manner, as both the H_2_O_2_- and rasfonin-induced, but not Rapa-induced autophagy, could be suppressed by inhibition of PFKFB3. The K472/473A mutated PFKFB3, which lost nuclear localizing property, inhibited the autophagic process associated with inhibition of the AMPK signaling. While H_2_O_2_ stimulated autophagy with upregulation of the activity of AMPK, either PFK-15 or rasfonin inhibited the H_2_O_2_-stimulated autophagic process concurring with downregulation of the activity of AMPK. Moreover, silencing of AMPK only inhibits the H_2_O_2_-induced, but not the rasfonin-induced autophagy.

In addition to its essential role in glycolytic flux induction, the nuclear targeted PFKFB3 has been demonstrated to promote cell proliferation [10], but whether its subcellular location regulates autophagy remains nearly unexplored. In rheumatoid arthritis T cells, PFKFB3 deficiency restrained activation of the autophagic process, whereas PFKFB3 inhibition promoted autophagy in HCT116 cells [11, 12]. Since either PFK-15 or depletion of PFKFB3 inhibited the H_2_O_2_- and rasfonin-induced autophagy, but neither suppressed the Rapa-dependent autophagic flux, we believe that PFKFB3 is likely to regulate autophagy in stimulus type-dependent manner. Importantly, subcellular localization of PFKFB3 has been demonstrated to play a regulatory role in autophagy. Although the K472/473A mutant stimulated more lactate production than the WT PFKFB3, it inhibited the autophagy induced by rasfonin or H_2_O_2_.

As an evolutionarily conserved energy-sensing kinase, AMPK is activated by metabolic stress or ATP consumption *in vivo*. Thus, it could also be linked to the regulation of autophagy, which is generally induced upon starvation. In addition to upregulation of AMPK by low cellular energy levels, some stimuli can also induce autophagy via activating AMPK under normal energy levels [42, 43]. It is well established that AMPK played a critical role to regulate autophagy, for example, to activate autophagy through inactivation of mTORC1 or direct phosphorylation of the protein kinase Ulk1 [19, 22]. Although being widely used as an inhibitor of AMPK, CC shows some special properties, such as inhibition of the activity of AMPK without decreasing the phosphorylation of AMPKα [40], and induction of autophagy by blocking the Akt/mTOR pathway [41]. Consistently, we observed that treatment with CC alone induced autophagy and inhibited the phosphorylation of ACC, but failed to decrease the phosphorylation of AMPKα. However, CC blunted the H_2_O_2_-induced autophagy accompanying by the attenuated phosphorylation of AMPKα and ACC. To avoid the aforementioned observations resulting from the off-target effects of CC, we also used the AMPK agonist AICAR and AMPKα1/2-target siRNAs to verify the results, and all supported that H_2_O_2_ induced autophagy by modulating the AMPK pathway. Interestingly, it was reported that inhibition of AMPK promoted autophagy in rat liver cells [24], here we observed that rasfonin induced autophagy accompanied by downregulation of the activity of AMPK. Rasfonin blunted the H_2_O_2_-upregulated activity of AMPK, and attenuated the autophagy induced by H_2_O_2_. Deprivation of AMPKα1/2 only inhibited the H_2_O_2_-, but not the rasfonin-induced autophagy. Thus, AMPK plays bidirectional roles in regulating autophagy, which may associate with the types of stimuli. Since both chemical and genetic inhibition of PFKFB3 dismissed the activity of AMPK, an inner connection between PFKFB3 and AMPK may exist in regulating autophagy.

Prior studies revealed that PFKFB3 was one of the downstream molecules of AMPK, and AMPK activated PFK-2 during ischemia to promote glycolysis [44, 45]. In addition, AMPK mitotic-specifically activates PFKFB3 at translational level, which protect cell survival during mitotic arrest [46]. However, PFKFB3 regulated autophagy by affecting the AMPK signaling, since either the K472/473A mutated PFKFB3 or deprivation of PFKFB3 reduced the activity of AMPK in response to H_2_O_2_ stimulation. While PFK-15 inhibited the basal and the H_2_O_2_-induced activities of AMPK, deprivation of PFKFB3 slightly increased the AMPK signaling. Expression of the K472/473A mutant reduced the phosphorylation of ACC, whereas the WT PFKFB3 increased the activity of AMPK in response to H_2_O_2_ stimulation. In the AMPKα1/2-silenced cells, rasfonin was demonstrated to stimulate autophagic process, which was attenuated by PFK-15, indicating that AMPK functions downstream of PFKFB3 under this circumstance. Thus, we speculated that the relationship between PFKFB3 and AMPK was likely to be cell type- and context-dependent, which was commonly found in different signaling pathways, such as crosstalk and compensation between Ras-Erk and PI3K-mTOR signaling [47]. Although it is well established that AMPK activates autophagy via direct phosphorylation of Ulk1 [23], Ulk1 was recently reported to mediate the phosphorylation of AMPK through a negative regulatory feedback loop [48]. Furthermore, AMPK was demonstrated to exert dual regulatory effects on the PI3K pathway [49]. Therefore, it is conceivable that PFKFB3 functions upstream of AMPK under certain circumstance. Considering the inhibitory effect of PFK-15 on the H_2_O_2_-induced autophagy in the AMPKα1/2-depleted cells, an effector other than AMPKα might function downstream of PFKFB3 to regulate the induced autophagic process. Although Rapa could activate AMPK [50], we found that inhibition of PFKFB3 showed no effect on the Rapa-induced autophagy, which may due to the fact that Rapa affected other signaling molecules, such as Akt and ERK [51].

In summary, the presented data clearly demonstrated that PFKFB3 showed the characteristics of nuclear localization, and the nuclear targeting of PFKFB3 promoted autophagy through the AMPK signaling pathway. In addition, the K472/473A mutation changed the subcellular localizing feature of PFKFB3 and failed to induce autophagy. These results shed light on the glycolytic pathway and autophagy regulation, and provide better understanding for the regulatory mechanism of autophagy.

## MATERIALS AND METHODS

### Chemicals and antibodies

Chloroquine diphosphate salt (CQ, C6628), bafilomycin A1 (Baf-A1, B1793), 1-(4-pyridinyl)-3-(2-quinolinyl)-2-propen-1-one (PFK-15, SML1009), Compound C (P5499), AICAR (A9978), 2-deoxyglucose (2-DG, D8375), and polyclonal antibodies against LC3 (L7543) were purchased form Sigma-Aldrich. Antibodies p44/42 MAPK (total-Erk1/2, 9102), PFKFB2 (13029), phospho-AMPKα (Thr172, 2535), total AMPKα (2532), phospho-ACC (Ser79, 3661), beta-tubulin (86298), and PARP-1 (9542) were purchased from Cell Signaling Technology. Antibodies of p62 (sc-28359) was acquired from Santa Cruz Biotechnology. Antibody of PFKFB3 (ab181861) was purchased from Abcam. Antibodies of Histone H3 (17168-1-AP) and Lamin B1 (12987-1-AP) were purchased from proteintech. Antibody against actin (TA-09) was obtained from ZhongShanJinQiao Biocompany. Alexa Fluor® 594 Goat anti-Rabbit IgG (H + L) secondary antibody (R37117) and Alexa Fluor® 488 Goat anti-Mouse IgG (H + L) secondary antibody (A-11001) were purchased from Molecular Probes. Rasfonin is stored in our lab.

### Plasmids and siRNAs

PFKFB3 plasmid was kindly provided by Dr. Yuqing Huo (Augusta University, GA, U.S.A.), and the K472/473A mutated PFKFB3 was constructed in our lab. The siRNA specific for human PFKFB3 (sc-44011), and AMPKα1/2 (sc-29673 and sc-38923) were purchased from Santa Cruz Biotechnology, along with the control siRNA (sc-37007).

### Cell culture and immunoblotting analysis

ACHN (human renal cancer) and HeLa (human cervical carcinoma cell line) cells were grown in DMEM media containing 10% fetal bovine serum (GIBCO) and antibiotics. Cells were grown to 70–80% confluency before adding the indicated compounds. Cells were treated in completed media containing 10% serum and collected at the indicted time points. For transfection, cells of 80% confluency were transfected using Lipofectamine 2000 (Invitrogen) or Attractene (QIAGEN) according to the manufacturer's protocol. After transfection for 24–36 h, cells were split and cultured overnight before subjecting to different treatments, immunoblotting, or analyzed by confocal microscopy.

For siRNA interference, cells of 30% confluence in their respective media without antibiotics were transfected using DharmaFECT (Dharmacon, T2001 or T2002) according to the manufacturer's instructions. Cells were split and cultured overnight before exposure to indicated treatment after transfection for 48 h. Triton X-100/glycerol buffer (containing 50 mM Tris-HCl, pH 7.4, 4 mM EDTA, 2 mM EGTA, and 1 mM dithiothreitol, supplemented with 1% Triton X-100, 1% SDS, and protease inhibitors) was used to acquire the whole cell lysates, which were then separated on a SDS-PAGE gel (13, 10 or 8% according to the molecular weights for the proteins of interest) and transferred to PVDF membrane. Immunoblotting was performed using appropriate primary antibodies and horseradish peroxidase-conjugated suitable secondary antibodies, followed by detection with enhanced chemiluminescence (Pierce Chemical).

### Subcellular fractionation

Cells were seeded into 100 mm-dishes at 70-80% confluency. After indicated treatment, cells were gathered, pelleted and washed three times with cold PBS. 20% cells were resuspended in Triton X-100/glycerol buffer and labeled as total homogenate (TH); the others were resuspended in 400 μl homogenization buffer A (10 mM Hepes-KOH [pH 7.9], 10 mM KCl, 1.5 mM MgCl_2_, 0.5 mM PMSF and 0.5 mM dithiothreitol) contained 0.5% NP-40, and then the homogenate was centrifuged at 3,000 rpm at 4°C for 5 min after static on ice for 15 min. Following wash twice with buffer A without NP-40, the pellet was resuspended in 60 ul buffer C (20 mM Hepes-KOH [pH 7.9], 600 mM KCl, 1.5 mM MgCl_2_, 0.2 mM EDTA and 25% glycerol). After rotating on ice for 15 min, the homogenate was centrifuged at 13,000 rpm at 4°C for 15 min, and the supernatant was collected as the nuclear fractions (Nu).

### Lactate assay

Cells were plated in 6-well plates, after the indicated treatments, suspension was collected and subjected to lactate assay. Levels of the secreted lactate were determined using the lactate assay kit (K-DATE, Megazyme). Data represent three independent experiments.

### Confocal/Fluorescence microscopy

Cells were plated on glass cover slips and the indicated treatments were performed. Cells were washed with Ca^2+^- and Mg^2+^-free PBS (CMF-PBS), fixed with freshly prepared 4% paraformaldehyde at 4°C for 30 min and permeabilized incubation with CMF-PBS containing 0.1% Triton X-100 and 0.5% BSA at room temperature (RT) for 5 min. Following washed three times with CMF-PBS, cells were blocked in CMF-PBS containing 0.5% BSA for 1 h, and incubated with the indicated antibodies in the presence of 0.1% Triton X-100 and 0.5% BSA. After washed three times, cells were incubated with the secondary antibodies (Alexa Fluor® 594 Goat anti-Rabbit and Alexa Fluor® 488 Goat anti-Mouse) diluted in CMF-PBS containing 0.5% BSA for 1 h. Cells were then immersed in VECTASHIELD with DAPI (H1200) to visualize the nuclei after washing for three times. Images were acquired via Confocal/Fluorescence microscopy.

### Electron microscopy

Briefly, samples were washed three times with PBS, trypsinized, and collected by centrifuging. Following fixed with 4% paraformaldehyde at 4°C overnight, cell pellets were post-fixed with 1% OsO_4_ in cacodylate buffer at RT for 1 h, and dehydrated stepwise with ethanol. The dehydrated pellets were rinsed with propylene oxide at RT for 30 min and embedded in Spurr resin for sectioning. For immunoelectron microscopy, the frozen cells were treated with 0.2% glutaraldehyde and 2% distilled water in acetone, and embedded in LR white. Ultrathin sections were stained with anti-PFKFB3 antibody, and secondary antibodies conjugated to 10-nm gold particles. Images of thin sections were observed under a transmission electron microscopy (JEM1230).

### Statistical analysis

Several X-ray films were analyzed to verify the linear range of chemiluminescence signals and quantifications were carried out using densitometry. Adjusted ratios of the indicated proteins to actin (A) and changed folds for levels of LC3-II and p62 after expose to CQ were presented below the blots. Normally distributed data are shown as mean ± SD and were analyzed using one-way analysis of variance and the Student-Newman-Keuls post-hoc test. Data are shown as Mean ± SD in Graphs.

## SUPPLEMENTARY MATERIALS FIGURES



## Abbreviations

ACC: acetyl-CoA carboxylase
AMPK: AMP-activated protein kinase
PFKFB3: 6-phosphofructo-2-kinase/fructose-2,6-bisphosphatase isoform 3
PFK-1: phosphofructokinase-1
PFK-15: 1-(4-pyridinyl)-3-(2-quinolinyl)-2-propen-1-one
Rapa: rapamycin
CC: Compound C
AICAR: 5-Aminoimidazole-4-carboxamide1-β-D-ribofuranoside
