# Supplementary material for: 6-Phosphofructo-2-kinase/fructose-2,6-bisphosphatase isoform 3 spatially mediates autophagy through the AMPK signaling pathway
Source: Oncotarget. 2017 Sep 8;8(46):80909–22. doi: 10.18632/oncotarget.20757 (PMC5655249; doi:10.18632/oncotarget.20757)
Supplement: Supplementary file 1 [file oncotarget-08-80909-s001.pdf]

6-Phosphofructo-2-kinase/fructose-2,6-bisphosphatase isoform 3 spatially mediates autophagy through the AMPK signaling pathway

SUPPLEMENTARY MATERIALS

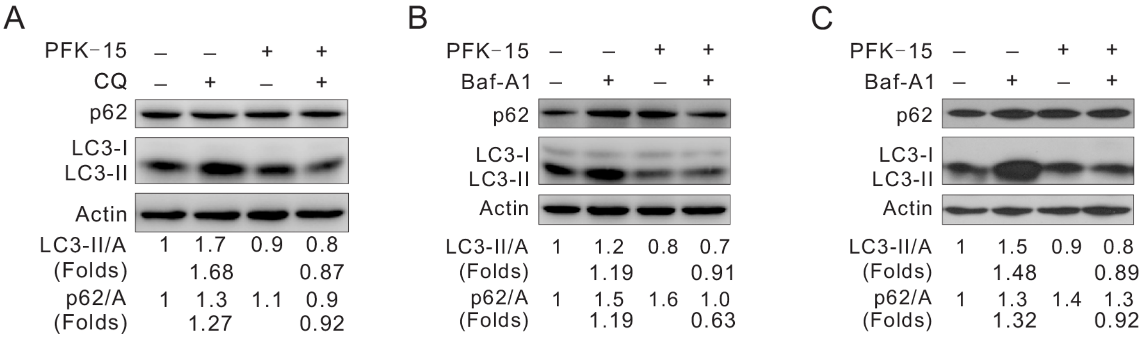

Supplementary Figure 1: PFK-15 attenuates autophagy. **(A)** HeLa cells were treated with 6 µM PFK-15 in the presence or absence of 10 µM CQ (A) or 10 nM Baf-A1 for 2 h. **(B** and **C)** HeLa and ACHN cells were treated with 6 µM PFK-15 in the presence or absence of 10 nM Baf-A1 for 2 h, respectively. Cell lysates were prepared and analyzed by immunoblotting using the indicated antibodies. The data represent three independent experiments.

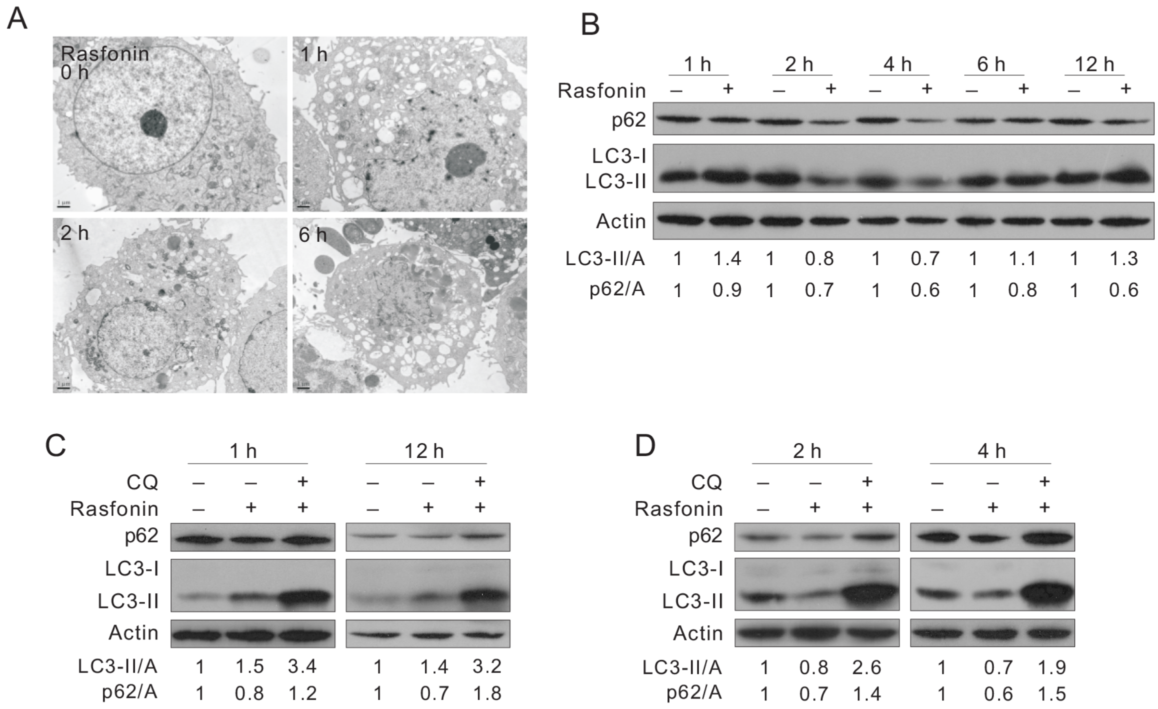

Supplementary Figure 2: Rasfonin is an autophagy inducer. (A) Electron microscopy was performed on ACHN cells following treatment with 6 μM rasfonin for up to 6 h. **(B−D)** ACHN cells were treated with 6 μM rasfonin in the presence or absence of 10 μM CQ for the indicated time points. Cell lysates were prepared and analyzed by immunoblotting using the indicated antibodies.

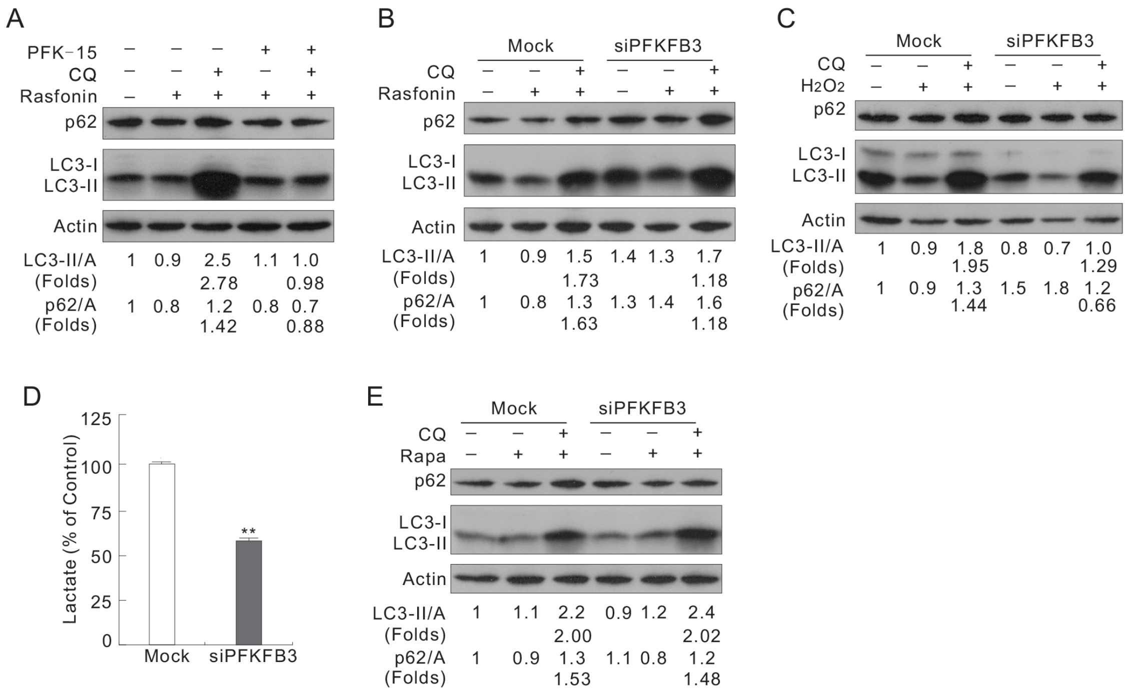

Supplementary Figure 3: Inhibition of PFKFB3 regulates autophagy in a stimulus type-dependent manner. **(A)** HeLa cells were treated with rasfonin or a combination with PFK-15 in the presence or absence of CQ for 2 h. **(B** and **D)** HeLa cells were transfected with the PFKFB3 siRNAs for 48 h. Following treatment with rasfonin with or without CQ for 2 h, cell lysates were analyzed by immunoblotting with the antibodies indicated (B); Suspensions were collected before treatments and performed lactate assay (D). Double asterisk means $p < 0.01$. **(C** and **E)** ACHN cells were transfected with the indicated siRNAs for 48 h. Following treatment with H_2O_2 or 0.1 μM Rapa with or without CQ for 2 h, cell lysates were analyzed by immunoblotting with the antibodies indicated. Similar experiments were repeated three times.

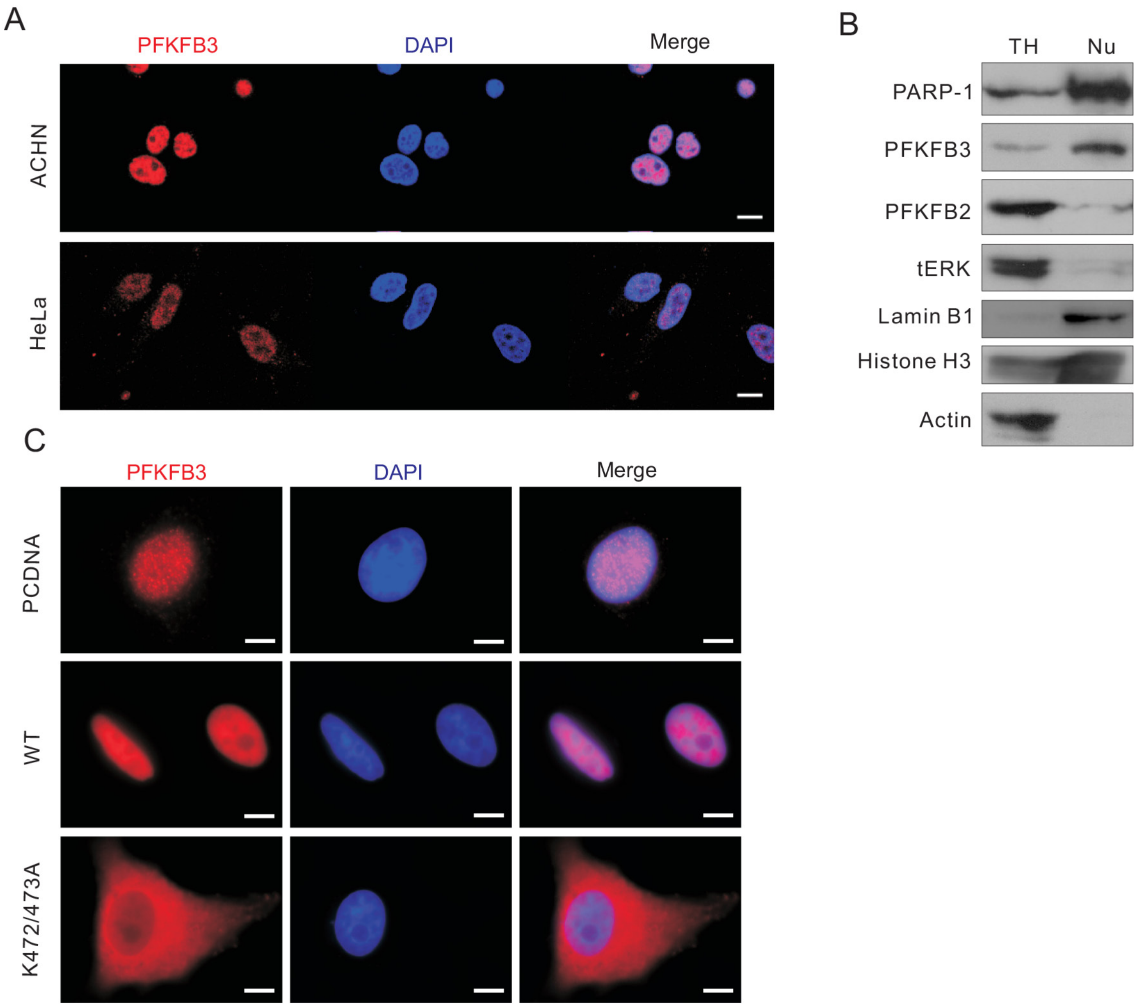

Supplementary Figure 4: Distribution of PFKFB3 is changed by K472/473A mutation. **(A)** ACHN and HeLa cells were stained by PFKFB3 antibody and DAPI, and pictured by confocal microscopy. Bar = 20 µm. **(B)** Total homogenate (TH) and nuclear fractions (Nu) were extracted from ACHN cells and analyzed by immunoblotting with the antibodies indicated. **(C)** HeLa cells were transfected with the WT or the K472/473A mutated PFKFB3 for 36 h. Cells were stained using the PFKFB3 antibody, and nuclei were stained by DAPI. Bar = 10 µm. Similar experiments were repeated twice.

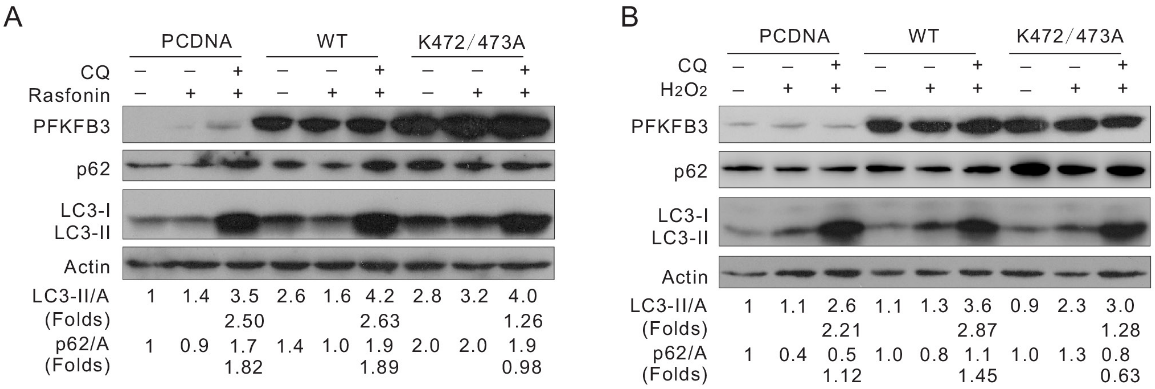

Supplementary Figure 5: Overexpression of the WT PFKFB3 promotes the induced autophagy. **(A)** HeLa cells were transfected with the indicated plasmids for 36 h, and treated with rasfonin in the presence or absence of CQ for 2 h. **(B)** ACHN cells were transfected with the indicated plasmids for 36 h, and treated with H_2O_2 in the presence or absence of CQ for 2 h. Cell lysates were analyzed by immunoblotting with the antibodies indicated. Similar experiments were repeated twice.

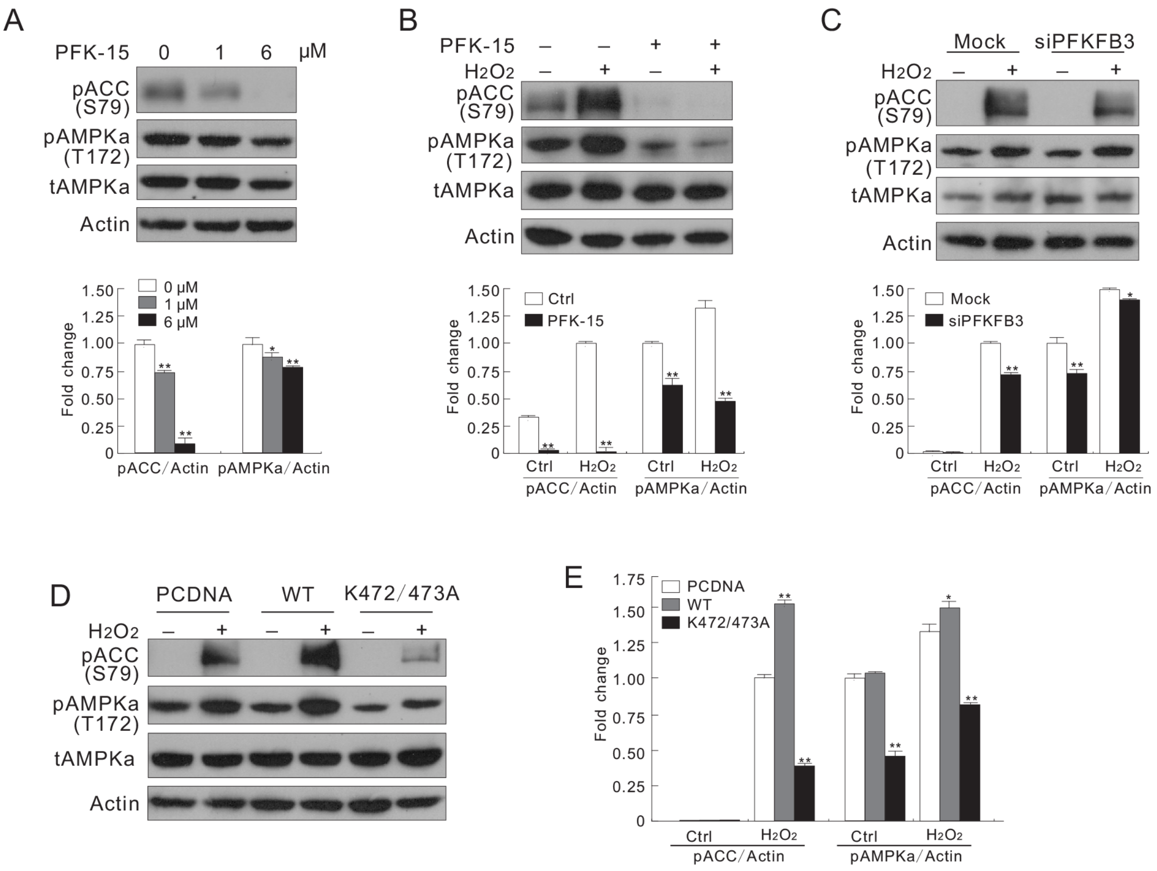

Supplementary Figure 6: AMPK plays a role in the PFKFB3-regulated autophagy. **(A)** HeLa cells were treated with 0–6 μM PFK-15 for 2 h. **(B)** ACHN cells were treated with PFK-15 in the presence or absence of H_2O_2 for 2 h. **(C)** ACHN cells were transfected with PFKFB3 siRNA for 48 h, and treated with H_2O_2 for 2 h. **(D and E)** Following transfection with the indicated plasmids for 36 h, ACHN cells were treated with H_2O_2 for 2 h. Cell lysates were analyzed by immunoblotting with the antibodies indicated. Quantification of the signals was shown the histogram (n = 3). *P < 0.05 vs. control; **P < 0.01 vs. control. Similar experiments were repeated twice.

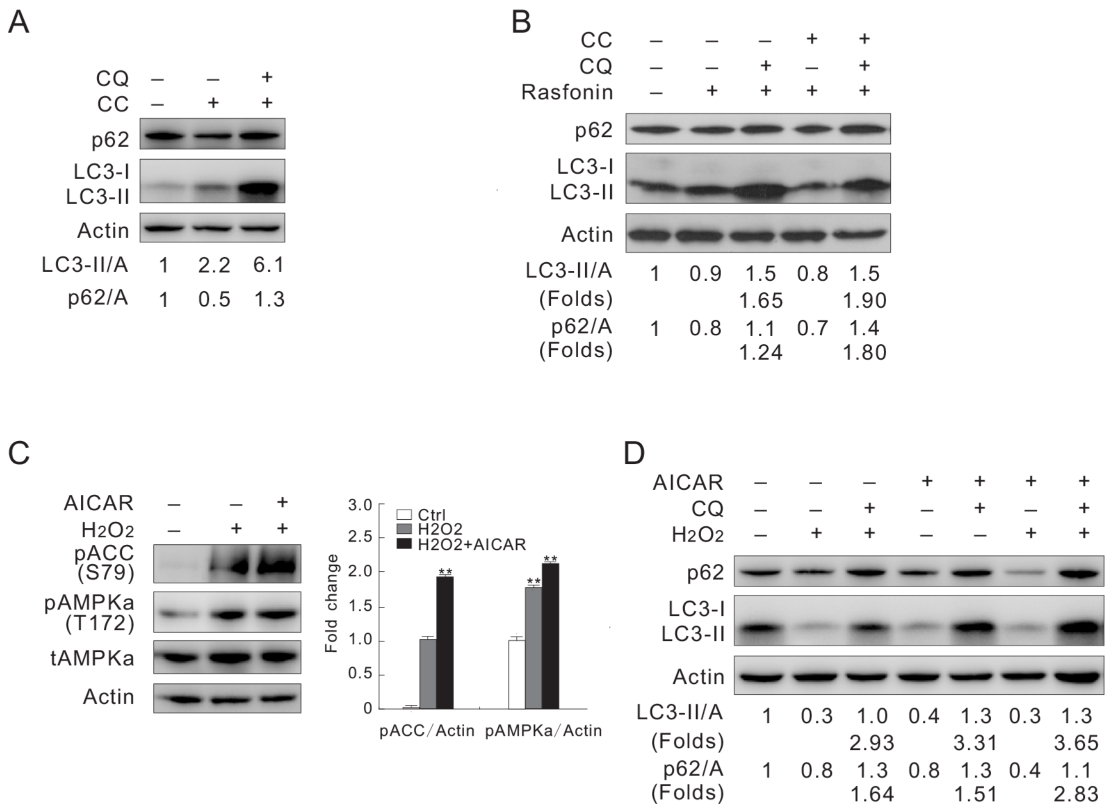

Supplementary Figure 7: AMPK agonist promotes the H_2O_2-activated autophagy. (A) ACHN cells were treated with 20 µM CC in the presence or absence of CQ for 2 h. **(B)** ACHN cells were treated with rasfonin or a combination with CC in the presence or absence of CQ for 2 h. **(C** and **D)** ACHN cells were treated with 2 mM AICAR or 0.1 mM H_2O_2 or a combination of both in the presence or absence of CQ for 2 h. Cell lysates were prepared and analyzed by immunoblotting using the indicated antibodies. Quantification of the signals were shown under the blot or in the bar graphs right (n = 3). **P < 0.01 vs. control. Similar experiments were repeated twice.
